# Characterization of Cyanobacterial Hydrocarbon Composition and Distribution of Biosynthetic Pathways

**DOI:** 10.1371/journal.pone.0085140

**Published:** 2014-01-27

**Authors:** R. Cameron Coates, Sheila Podell, Anton Korobeynikov, Alla Lapidus, Pavel Pevzner, David H. Sherman, Eric E. Allen, Lena Gerwick, William H. Gerwick

**Affiliations:** 1 Center for Marine Biotechnology and Biomedicine, Scripps Institution of Oceanography, University of California San Diego, La Jolla, California, United States of America; 2 Skaggs School of Pharmacy and Pharmaceutical Sciences, University of California San Diego, La Jolla, California, United States of America; 3 Algorithmic Biology Laboratory, St. Petersburg Academic University, Russian Academy of Sciences, St. Petersburg, Russia; 4 Department of Mathematics and Mechanics, St. Petersburg State University, St. Petersburg, Russia; 5 Theodosius Dobzhansky Center for Genome Bionformatics, St. Petersburg State University, St. Petersburg, Russia; 6 Department of Computer Science and Engineering, University of California San Diego, La Jolla, California, United States of America; 7 Life Sciences Institute and Department of Medical Chemistry, University of Michigan, Ann Arbor, Michigan, United States of America; Brandeis University, United States of America

## Abstract

Cyanobacteria possess the unique capacity to naturally produce hydrocarbons from fatty acids. Hydrocarbon compositions of thirty-two strains of cyanobacteria were characterized to reveal novel structural features and insights into hydrocarbon biosynthesis in cyanobacteria. This investigation revealed new double bond (2- and 3-heptadecene) and methyl group positions (3-, 4- and 5-methylheptadecane) for a variety of strains. Additionally, results from this study and literature reports indicate that hydrocarbon production is a universal phenomenon in cyanobacteria. All cyanobacteria possess the capacity to produce hydrocarbons from fatty acids yet not all accomplish this through the same metabolic pathway. One pathway comprises a two-step conversion of fatty acids first to fatty aldehydes and then alkanes that involves a fatty acyl ACP reductase (FAAR) and aldehyde deformylating oxygenase (ADO). The second involves a polyketide synthase (PKS) pathway that first elongates the acyl chain followed by decarboxylation to produce a terminal alkene (olefin synthase, OLS). Sixty-one strains possessing the FAAR/ADO pathway and twelve strains possessing the OLS pathway were newly identified through bioinformatic analyses. Strains possessing the OLS pathway formed a cohesive phylogenetic clade with the exception of three *Moorea* strains and *Leptolyngbya sp.* PCC 6406 which may have acquired the OLS pathway via horizontal gene transfer. Hydrocarbon pathways were identified in one-hundred-forty-two strains of cyanobacteria over a broad phylogenetic range and there were no instances where both the FAAR/ADO and the OLS pathways were found together in the same genome, suggesting an unknown selective pressure maintains one or the other pathway, but not both.

## Introduction

Cyanobacteria are a diverse group of photosynthetic bacteria that have evolved a remarkable array of adaptive traits including oxygenic photosynthesis, N_2_ fixation, a wide morphological diversity, extensive secondary metabolite biosynthetic capacity, and a range of symbiotic relationships with other organisms. Cyanobacteria are estimated to contribute 30% of Earth's annual oxygen production and play a major role in biogeochemical cycles [1]. One trait less well characterized and potentially of great societal importance is their universal ability to produce long chain hydrocarbons. First recognition of this latter trait resulted from investigations in the 1960's [2]–[4] and was of importance in the context of identifying the origin of hydrocarbons found in sedimentary and oil deposits. In recent years, there has been a growing recognition of the negative environmental impacts of continued fossil fuel use, as well as an ever increasing worldwide energy demand, and these facts have combined to increase interest in developing sustainable biofuels such as hydrocarbons from cyanobacteria [5], [6]. Many sources of renewable energy can be envisioned to help meet society's demand for electrical power, however, there remains an acute need for low cost liquid fuels, and particularly gasoline, diesel and jet fuel [6].

Diesel and jet fuel quality are measured using cetane values whereas octane ratings are the principal measure of gasoline quality. Quality of the fuel is thus largely determined by the structure of the hydrocarbons in the fuel, and the type of fuel being considered. Long, straight-chain saturated hydrocarbons exhibit higher cetane ratings whereas short highly branched molecules (i.e. isooctane) exhibit superior octane ratings [7], [8]. It is important to consider these structural characteristics when assessing the applicability of various biofuel molecules to different engine applications. In this context, the commonly observed hydrocarbons of cyanobacteria, including heptadecane (cetane rating: 105) and methylheptadecane (cetane rating: 66), are promising candidates for diesel fuel applications (normal cetane rating: 40–55) [5], [7], [8].

In a broader context, cyanobacteria are one of only a few types of organisms that are known to directly produce hydrocarbons. However, their highest reported native production of alk(a/e)nes is 0.12% of dry biomass [4], and the physiological or ecological function of alk(a/e)ne production in cyanobacteria is not yet understood. In this latter regard, various possibilities exist including prevention of grazing from herbivores, intra- or inter- species chemical signaling, prevention of desiccation, enhanced buoyancy, or membrane fluidity/stability. Unfortunately, none of these potential functions have been directly evaluated, and this remains an area in need of further investigation.

It is important to recognize that the extremely low native yields of hydrocarbons from wild type cyanobacterial strains are insufficient to be cost competitive with petroleum derived fuels, and therefore, some moderation of the expression of cyanobacterial hydrocarbons pathways will be necessary if they are to be used in an industrial or societal context. Thus, a deeper understanding of these pathways is needed in order to rationally engineer and enhance this trait in modified organisms.

Fatty acid-derived hydrocarbons are produced in a variety of life forms. As described more extensively below, cyanobacteria synthesize long chain alk(a/e)nes using two different pathways, one of which involves a deformylation of fatty aldehydes and the other decarboxylation of fatty acids [2], [5], [9]. Other prokaryotes such as *Jeotgalicoccus sp.* biosynthesize alk(a/e)ne through cytochrome P450-catalyzed decarboxylation of fatty acids to form terminal olefins [10]. Long-chain alkenes in *Micrococcus luteus* ATCC 4698 are formed via head-to-head condensation of fatty acids [11]. Although some of the final products from these hydrocarbon pathways are identical to the alk(a/e)nes produced by cyanobacteria, the specific biosynthetic steps involved are distinctly different.

Hydrocarbons produced via fatty acid pathways have also been described in various eukaryotic organisms including the long-chain fatty acid derived alkenes from *Botryococcus braunii* race A [12], [13], the pheromone attractant hormosirene in the diatom *Gomphonema parvulum* [14], and the pheromone 7-methylheptadecane found in lepidopteran insects [15], [16]. However, these eukaryotic hydrocarbons are derived from entirely different biosynthetic pathways than cyanobacterial hydrocarbons. For example, the *Botryococcus braunii* race A alkenes are thought to derive from an aldehyde decarbonylase, hormosirene biosynthesis in the diatom *Gomphonema parvulum* involves a hydroperoxide lyase, and a P450 has recently been described as responsible for fatty acid decarbonylation to produce branched alkanes in lepidopteran insects.

**Figure 1:**
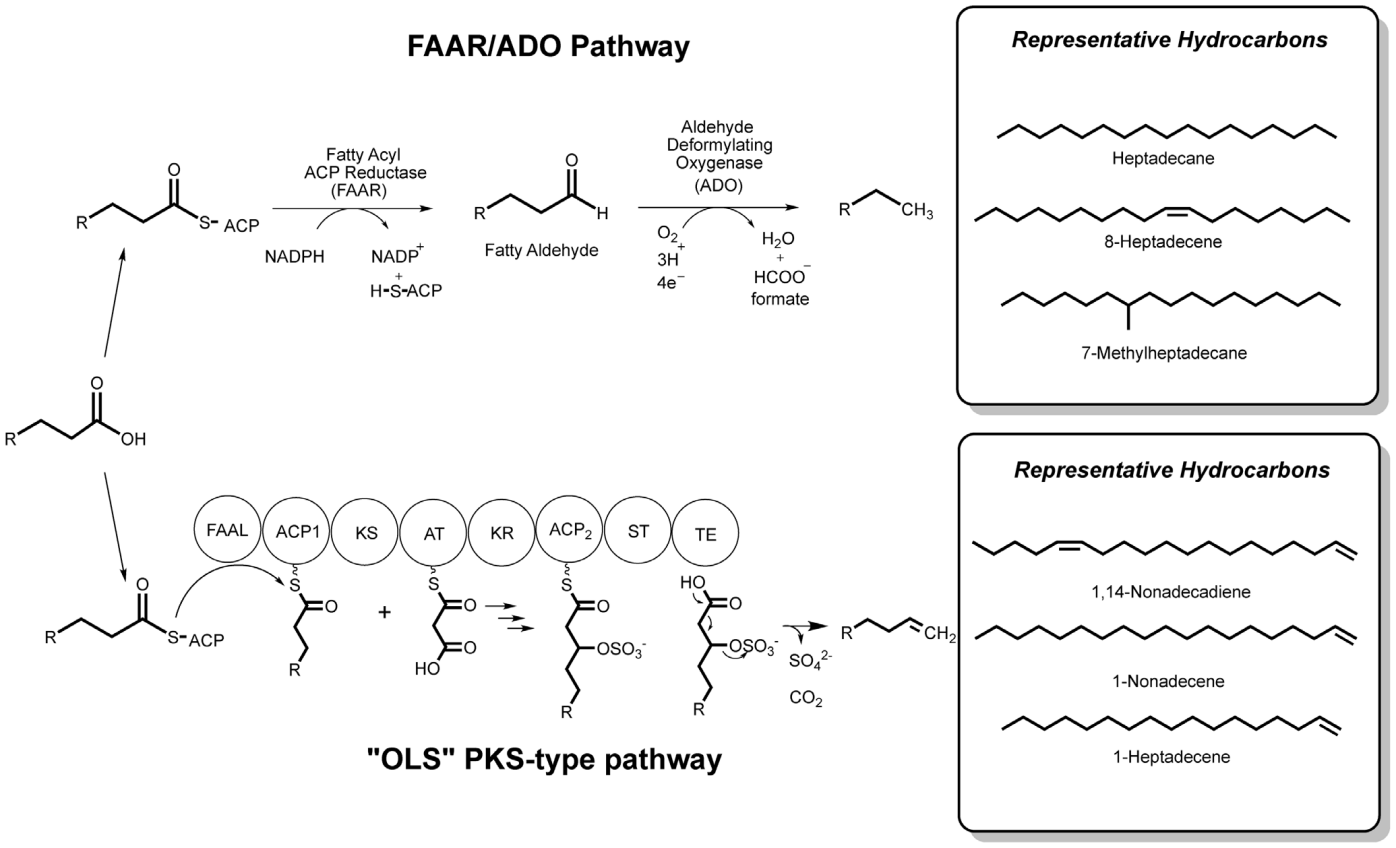
Hydrocarbon biosynthetic pathways in cyanobacteria. A) The Fatty Acyl-ACP Reductase (FAAR)/Aldehyde Deformylating Oxygenase (ADO) involves first a reduction of a fatty acyl substrate to a fatty aldehyde followed by an oxidative conversion to an alkane with the release of formate [17]. The OLS (olefin producing) pathway involves a polyketide synthase that first elongates a fatty acyl-CoA by two carbons from malonyl-CoA via ketosynthase (KS) and acyl transferase (AT) domains followed by reduction to the β-hydroxyacid by a ketoreductase (KR). The ST activates the β-hydroxy group via sulfonation, and then the thioesterase (TE) acts on this substrate to catalyze decarboxylation and loss of sulfate to form the terminal alkene.

Within just the last few years, two separate pathways have been described in cyanobacteria that produce hydrocarbons from fatty acid substrates, with each pathway involving a quite different mechanism for removing the terminal oxidized carbon atom [5], [9]. One pathway involves the conversion of fatty acyl-ACPs to fatty acyl-aldehydes by a fatty acyl-ACP reductase (FAAR), followed by a unique oxygen dependent conversion of the aldehyde via an aldehyde-deformylating oxygenase (ADO) to produce an odd-chain length saturated alkane (or alkene if the fatty acid possesses preexisting unsaturation) (Figure 1) [5], [17]). FAARs have been found in a wide range of organisms including plants, eukaryotic algae, bacteria and humans [18]. This reaction is known to require NADPH that likely provides a hydride that attacks the carbonyl of the fatty acid thioester. Nevertheless, the precise mechanism of this FAAR enzyme has not been conclusively verified [5] and there are no published crystal structures of a FAAR enzyme from any source. The next step involving the ADO enzyme is unique to cyanobacteria [17]. This enzyme was first described by [5] as an aldehyde decarbonylase after verifying its function through mutagenesis, heterologous expression, and a re-investigation of a previously published crystal structure of the ADO enzyme from *Prochlorococcus marinus* MIT9313. Carbon monoxide was initially considered to be the oxidized byproduct of this reaction [5], [19]. However, after some debate, it was shown by Li *et al.* to produce an alkane and formate from a unique oxygenation reaction thus justifying an alternative name of aldehydes deformylating oxygenase [17].

In 2010, Ridley *et al.* [20] and subsequently in 2011, Mendez-Perez *et al.* [9] and Donia *et al.* [21] published reports on terminal olefin biosynthetic pathways in *Synechococcus sp.* PCC 7002 and *Prochloron didemni*. Mendez-Perez verified the function of the OLS pathway in *Synechococcus sp.* PCC 7002, tested a variety of potential substrates, and enhanced productivity using an alternative promoter [9]. Recently, Gehret-McCarthy *et al.* compared the structure and function of the activating sulfotransferases (STs) from the CurM pathway in *M. producens* 3L and the OLS pathway in *Synechococcus sp.* PCC 7002, and found that both STs accepted substrates leading to production of long-chain hydrocarbons [22]. This second described pathway in cyanobacteria thus uses fatty acid substrates and distinctively produces odd-numbered carbon chains with terminal olefinic bonds. In 2011, Donia *et al.* and Mendez-Perez *et al.* identified a large (∼10 Kb) multi-domain PKS pathway that proceeds via an elongation/decarboxylation mechanism (Figure 1) [21], [9]. The pathway was initially located by searching for homologs of the well characterized CurM domain of the curacin A biosynthetic pathway in *Moorea producens* 3L (formerly *Lyngbya majuscula* 3L) [9], [21], [23], [24]. Curacin A is a secondary metabolite with promising anticancer activity [25]. CurM is known to produce the terminal alkene functionality in curacin A via an elongation-decarboxylation mechanism and has recently been shown to accept fatty acid substrates to produce terminal alkenes [22], [25]. Mendez-Perez *et al.* proposed that the olefin-producing pathway (the OLS pathway) includes an ATP-consuming fatty acyl ACP ligase (FAAL) domain that transfers a fatty acid or fatty acyl-ACP to the OLS acyl carrier protein (ACP) [9]. The ACP-bound substrate subsequently undergoes elongation by an extension module [ketosynthase (KS), acyltransferase (AT), and ketoreductase (KR)] to add two carbons from malonyl-CoA and reduce the β-keto group to a β-hydroxy functionality. Gehret-McCarthy *et al.* characterized the final step of the OLS pathway to involve a sulfotransferase (ST) and thioesterase (TE) that transfers sulfonate from the donor 3′-phosphoadenosine-5′-phosphosulfate (PAPS) to the β-hydroxyacyl substrate [22]. This activates the intermediate for subsequent TE-catalyzed hydrolysis from the enzyme surface, decarboxylation and desulfation to form the terminal alkene product [22]. The inherent formation of a terminal double bond in the product is a unique structural signature of the OLS pathway compared to the FAAR/ADO pathway (Figure 1).

**Figure 2:**
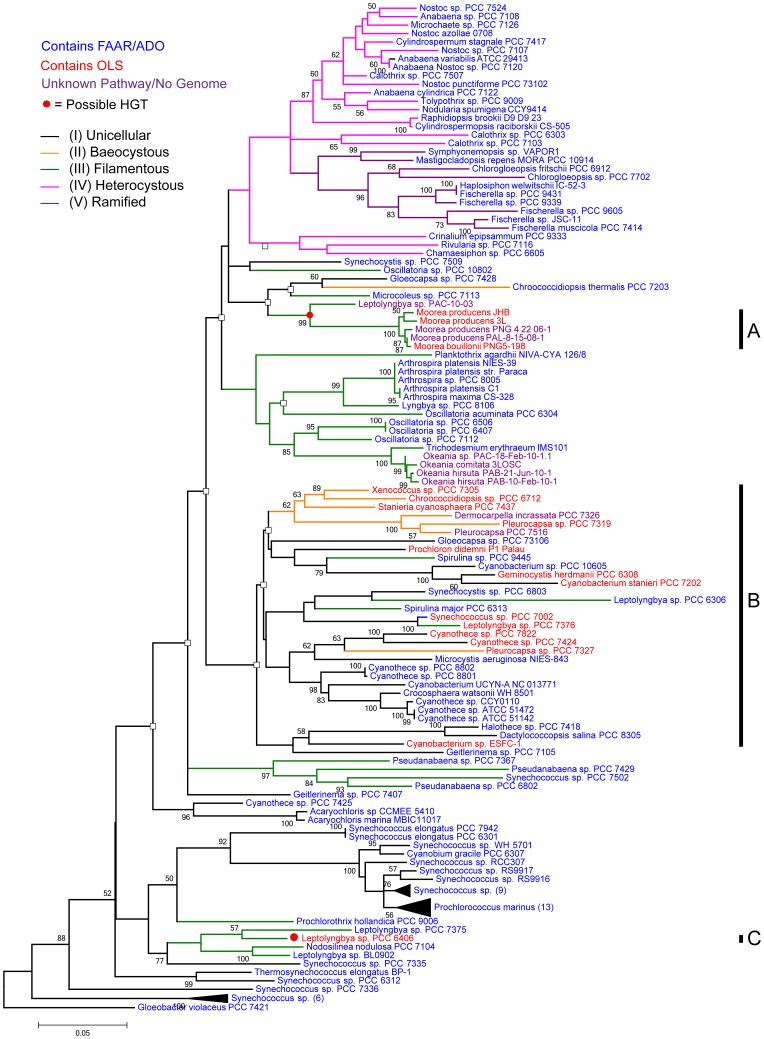
Cyanobacterial 16S rRNA phylogeny and hydrocarbon pathway distribution. The 16S rRNA phylogeny of publicly available genome sequenced cyanobacteria (128) and additional strains investigated in this study (14) including *G. violaceus* PCC 7421 as the outgroup. Blue strain names indicate strains possessing the FAAR/ADO pathway and red indicates those with the OLS pathway. Purple strain names indicate a strain that does not have a genome sequence and therefore pathway presence cannot yet be verified. Cyanobacterial subdivisions are labeled using colored branches following the key in the upper left: (1) Subdivision 1. Uniceullular (Formerly Chroococcales), Subdivision II. Baeocystous (Formerly Pleurocapsales), Subdivision III. Filamentous (Formerly Oscillatoriales), Subdivision IV. Heterocystous (Formerly Nostocales), Subdivision V. Ramified or True Branching (Formerly Stigonematales). The clade indicated as “A” represents the main clade of cyanobacteria with the OLS pathway while clade “B” indicates the *Moorea* strains and clade “C” indicates the clade containing *Leptolyngbya sp.* PCC 6404. Baysian posterior probabilities are displayed at nodes (o = posterior support<0.5).

Although these two cyanobacterial hydrocarbon biosynthetic pathways were only recently identified and characterized, knowledge of the diagnostic structural features of the resulting alk(a/e)nes and their respective gene sequences can be used along with previous reports of cyanobacterial hydrocarbon compositions to better understand the taxonomic distribution of these two pathways and the structural diversity found in cyanobacterial hydrocarbons (Table S1). However, these previous investigations have focused on a small subset of cyanobacteria, mostly comprised of strains from subdivision 1 (Unicellular), subdivision III (Filamentous) and subdivision IV (Heterocystous); only a very limited number of investigations on the hydrocarbons of subdivision II (Baeocystous) and subdivision V (Ramified) have been conducted (Table S1) [26]. In this context, the older morphology-based taxonomic perspective of cyanobacteria is severely limited due to the complex evolution of multicellularity in cyanobacteria. Indeed, 16S rRNA-based phylogenetic trees of the cyanobacteria reveal that the cyanobacterial subdivisions are polyphyletic [27] (Figure 2, Table S2). Nonetheless, the limited sampling to date of cyanobacterial diversity for hydrocarbons suggested that there may be as yet uncharacterized structural diversity. Additionally, previous reports of cyanobacterial hydrocarbons have rarely verified chain length, double bond positions or methyl group positions with authentic standards or other rigorous methods of structural analysis, but rather, have relied heavily upon mass spectral library searches (Table S1) [28], [29]. This latter approach introduces ambiguity concerning these key structural features, and therefore, could contribute to misunderstandings concerning the nature and distribution of hydrocarbon biosynthetic pathways and their evolution. For example, a number of the reported hydrocarbons are inconsistent with either of the known mechanisms of hydrocarbon production, such as naturally derived even chain-length hydrocarbons [30]–[33] despite the expected production of only odd-chain alk(a/e)nes from either the FAAR/ADO or OLS pathways following predicted and the experimentally verified pathway mechanisms [5], [9], [22]. This observation could be explained by either the existence of yet another pathway for alk(a/e)ne biosynthesis, or that the reported even chain alk(a/e)nes have been mis-identified.

The current study thus aimed to expand the characterization of structural diversity of cyanobacterial hydrocarbons through rigorous structural identification methods and across a wide range of cyanobacterial diversity. We hypothesized that this expanded structural diversity may reveal new insights concerning cyanobacterial biosynthetic pathways. Additionally, through a comparison of the hydrocarbon pathways found in the currently available sequenced cyanobacterial genomes, we were able to develop a deeper understanding of the relationships and evolution of these biosynthetic pathways in cyanobacteria.

## Materials and Methods

### Strain Selection

Strains of cyanobacteria were selected from a wide phylogenetic distribution ensuring that every major clade was sampled (Figure 2). Strains of cyanobacteria with available genome sequences and established genetic techniques were given preference. Strains that required extremely low or high temperature or unusual media requirements for growth were excluded. *Anabaena (Nostoc) sp.* PCC 7120 was provided by James Golden at UCSD. *Synechococcus elongatus* PCC7942 and *Leptolyngbya* BL0902 was provided by Susan Golden at UCSD. *M. producens* 3L, *Moorea bouillonii* PNG5-198 and *Moorea producens* JHB, *Leptolyngbya sp.* PAC10-3, *Moorea sp.* PNG 4/22/06-1, *Moorea sp.* PAL8/15/08-1, *Okeania comitata* 3LOSC, *cf. Okeania hirsuta*. PAB 10-Feb-10-1, *Okeania hirsuta* PAB 21-Jun-10-1, *cf. Phormidium sp.* ISB 3/Nov/94-8, *Okeania sp.* PAC-18-Feb-10-1.1 were isolated from field collections and are maintained among the Gerwick Laboratory culture collection as described in [24], [34], [35]. *Planktothrix agardhii* NIVA-CYA 168 was provided by Rainer Kurmayer at the Austrian Academy of Sciences Institute for Limnology. *Haplosiphon welwitchii* IC-52-3 and *Westiella intricata* HT-29-1 were provided by Thomas Hemscheidt of University of Hawaii via Melinda Micallef and Michelle Moffitt. The remaining strains were purchased from the Pasteur Culture Collection of Cyanobacteria (PCC) or the American Type Culture Collection (ATCC) as identified in the text.

### Culture Conditions

Cyanobacterial strains were grown in 2.8 L Fernbach flasks under 16∶8 day: night light cycle between 20–60 µE/m^2^/sec at a constant temperature at 20°C, 25°C, or 28°C in BG-11, SWBG-11, or ASNIII. Cultures were shaken continuously (80 rpm) or grown statically between 14 and 35 days depending upon strain growth rates. Cultures (1L) were harvested via centrifugation at 4000 rpm for 15 min in 500 mL conical containers and combined (using 0.5 M ammonium formate to remove salts for marine strains and deionized water for freshwater strains) into 50 mL Falcon tubes and subsequently centrifuged again to yield a packed pellet that was frozen and dried for extraction and analysis. For filamentous strains that were not amenable to centrifugation, filaments were removed from the media using forceps (rinsed using 0.5 M ammonium formate to remove salts for marine strains), and frozen for drying, extraction and analysis. All biomass was lyophilized for at least 24 h.

### Extraction and Structural Analysis

Dried biomass was ground using a mortar and pestle and weighed. Biomass was extracted using 5 mL of 100% hexanes or 2∶1 dichloromethane∶methanol (DCM∶MeOH) followed by 20 sec of sonication. The extract was filtered using Whatman GF/F and the residual biomass re-extracted two additional times using the same method followed by a 10 mL wash with hexanes or 2∶1 DCM∶MeOH. Octadecane (Fluka-74691) was added to each extract after initial solvent addition as an internal standard for quantitation. Octadecane was added at approximately 0.1% of the initial dry biomass. DCM∶MeOH (2∶1) crude extracts were fractionated using a normal phase 500 mg Bonna-Agela Cleanert silica SPE column with collection of the first fraction which eluted with 100% hexanes. Extracts were dried under N_2_ gas. A comparison between hexane and DCM∶MeOH extraction methods for three strains (*Anabaena (Nostoc)* PCC 7120, *S. elongatus* PCC 7942, *Synechococcus sp.* PCC7002) found that yields and composition of hydrocarbons were not significantly different (data not shown).

To characterize potential fatty acid substrates for hydrocarbon biosynthesis, a fatty acid analysis was performed on crude extracts (2∶1 DCM∶MeOH) of *Anabaena (Nostoc) sp.* PCC7120, *M. producens* 3L, and *Synechococcus sp.* PCC 7002. Fatty acid methyl esters (FAMEs) were produced by transesterification by adding 3 mL of 4% H_2_SO_4_ (in MeOH) to at least 0.1 mg of a crude extract. Samples were then stirred and incubated at 110°C for 1 h. Four mL of H_2_O and 3 mL of hexanes were then added to the sample. After vortexing for 30 sec and centrifugation at 2500 rpm for 3 min the hexanes layer (top) was removed and dried in a pre-weighed vial for GC-MS analysis. FAME preparation was followed by dimethyl disulfide (DMDS) derivitization for determination of double bond positions [36].

Each extract was resuspended to a concentration of 100 µg/mL in hexanes and 1 µL was analyzed by gas chromatography mass spectrometry (GC-MS) using a Thermo Trace GC-DSQ instrument equipped with an Agilent DB5-ms column (30 m, ID: 0.25, Film: 0.25 µm). Helium (constant flow 1 mL/min) was used as the carrier gas. The inlet temperature was 240°C and the following temperature program was applied: 40°C for 1 min with an increase of 4.5°C/min to 250°C for 10 min. Data were acquired and processed with the Thermo Xcaliber software. Hydrocarbons were determined using a combination of mass fragmentation patterns, retention time and comparison to authentic standards when available [heptadecane (Fluka-51578), 1-heptadecene (TCI-S0347), 7-methylheptadecane (kindly provided by Dieter Enders and Wolfgang Bettray, RWTH, Aachen University)], or published mass spectra and the NIST mass spectral library for Xcaliber (2005) when not available. Double bond positions were confirmed for all alkenes and unsaturated fatty acids using the DMDS method [36].

GC-MS detector response factors for heptadecane, 1-heptadecene, and 7-methylheptadecane were determined in comparison with the octadecane standard by creation of standard curves. Standard curves for hydrocarbons with authentic standards were verified using the low mass common to all hydrocarbons analyzed via GC-MS (57 *m/z*). Hydrocarbon concentration was calculated using hydrocarbon peak area compared to the internal standard (octadecane) peak area using 57 *m/z*. Percent dry weight was calculated as an average of three biological replicates. Statistical analysis of the hydrocarbon yields between the OLS and FAAR/ADO pathway were completed using a Mann-Whitney-Wilcox Test.

### Genome Sequencing and Bioinformatic Analysis

The sequenced genomes of *M. bouillonii* PNG5-198, *M. producens* JHB, and *cf. Phormidium sp.* ISB 3/Nov/94-8 were generated at either the Genomic Center at The Scripps Research Institute or at the University of Michigan using Illumina technology [37]. Genomes were corrected with Quake and assembled using SPAdes 2.4 [38], [39]. Contigs were binned by GC content to remove non-cyanobacterial DNA sequences.

Identification of cyanobacterial hydrocarbon pathways was accomplished using blastn searches against newly sequenced genomes and blastp searches using representative genes from each pathway (FAAR/ADO and OLS) against publicly available cyanobacterial genomes (131 total) from GenBank [40] and the Joint Genome Institute (JGI) Integrated Microbial Genomes (IMG) database (Version 4, [41]. Draft genome sequences for *M. bouillonii* PNG5-198, *M. producens* JHB, *cf. Phormidium sp.* ISB 3/Nov/94-8, *P. agardii* NIVA-CYA 126/8 (provided by Rainer Kurmayer, bio project accession number: PRJNA163669), *H. welwitschii* IC-52-3 and *W. intricata* HT-29-1 (provided by Melinda Micallef and Michelle Moffitt), and *Leptolyngbya sp.* BL0902 (provided by Arnaud Taton, Susan Golden and James Golden) were also used. The sulfotransferase domain in the OLS pathway identified in *Synechococus sp.* PCC 7002 was used for this search because this is a distinctive enzymatic step in terminal alkene biosynthesis [22]. The FAAR and ADO enzymes from *S. elongatus* PCC 7942 were used as representative sequences in the search for FAAR/ADO pathways. Searches for FAAR/ADO pathway enzymes were also performed using hidden Markov model protein family patterns TIGR04058 (aldehyde-forming long-chain fatty acyl-ACP reductase) and TIGR04059 (long-chain fatty aldehyde decarbonylase) from the TIGRFAMs database [42].

### Phylogenetic Analysis

To establish and compare the phylogenetic distribution of hydrocarbon biosynthetic pathways in cyanobacteria, phylogenies were constructed using the 16S rRNA gene sequences from all cyanobacterial strains for which genome sequence data is publically available (128) as well as all additional cyanobacteria investigated in this study (16). Due to incomplete or partial sequences, *cf. Phormidium sp.* ISB 3/Nov/94-8, *W. intricata* HT-29-1, and *Synechococcus sp.* CB0205 were omitted from these analysis. *Pleurocapsa sp.* PCC 7320, *Cyanobacterium sp.* JSC-1 as well as *Synechococcus sp.* CB0101 were not included in the phylogenetic analyses because 16S rRNA sequences are not currently available for these strains. Alignments of the 16S rRNA sequences were completed using MAFFT and trees were generated using both PhyML (GTR+I+G substitution model, 500 bootstrap replicates) and MrBayes (GTR+I+G substitution model). OLS pathway amino acid sequences were recovered as described above and aligned using MAFFT and trees were generated using PhyML (LG substitution model, 500 bootstrap replicates).

## Results

### Hydrocarbon Composition

**Figure 3:**
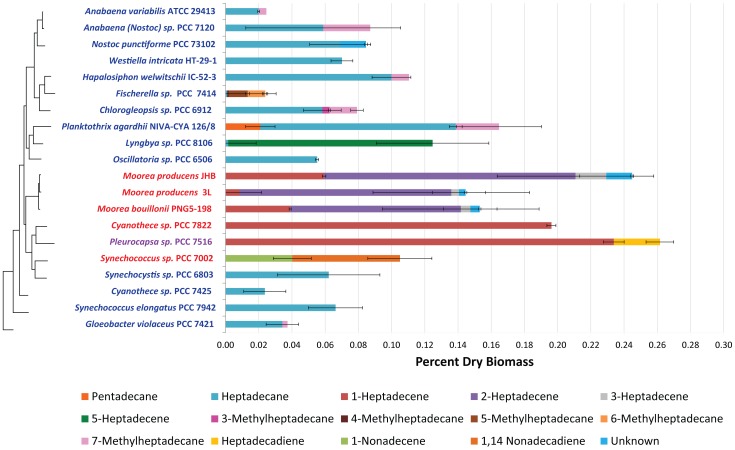
Quantitative yields of hydrocarbons as percent dry weight of biomass from 20 cyanobacteria displayed by phylogenetic relationship and pathway distribution. Specific hydrocarbons for each strain are color coded and stacked to depict the overall quantitative yield. Standard error bars are given for each hydrocarbon and each strain. Quantitative hydrocarbon yields ranged from 0.024%±0.01% in *Cyanothece sp.* PCC 7425 to 0.262%±0.01% in *Pleurocapsa sp.* PCC 7516. Blue strain names indicate strains possessing the FAAR/ADO pathway and red indicates those with the OLS pathway. Purple strain names indicate a strain that does not have a genome sequence and therefore the pathway type is unknown. To the left of the figure, a 16S rRNA phylogenetic tree (Maximum Likelihood, see Figure S1 for complete tree) is presented for all 20 displayed strains. Branch tips are aligned to corresponding strain names except for *Westiella intricata* HT-29-1 for which no 16S rRNA sequence is available. Vertical connection lengths were modified to accommodate the location of *W. intricata* HT-29-1 in the table.

The hydrocarbon composition of thirty-two cyanobacterial strains were analyzed and compared in the context of their respective biosynthetic pathways. Hydrocarbon yields as a percent of dry biomass ranged from 0.024%±0.01% in *Cyanothece sp.* PCC 7425 to 0.262%±0.01% in *Pleurocapsa sp.* PCC 7516 (Figure 3 and S1), and averaged 0.11% (±0.015%) across all strains. Strains possessing the OLS pathway appear to have significantly higher hydrocarbon yields (0.173%±0.032%) than strains with the FAAR/ADO pathway (0.070%±0.008%) (p-value = 0.0002). Heptadecane was the most commonly observed hydrocarbon (24 out of 32 strains). Alkanes such as heptadecane as well as all branched alkanes (e.g. 7-methylheptadecane), were only observed in strains that contained the FAAR/ADO pathway while strains with the OLS pathway appeared to only produce alkenes. *Lyngbya sp.* PCC 8106 is the only strain that produced alkenes and also possessed the FAAR/ADO pathway. However, *Lyngbya sp.* PCC 8106 produced 5-heptadecene, an alkene with an internal double bond instead of a terminal alkene consistent with the OLS pathway. Therefore, 5-heptadecene in *Lyngbya sp.* PCC 8106 is likely derived from a monounsaturated fatty acid.

**Figure 4:**
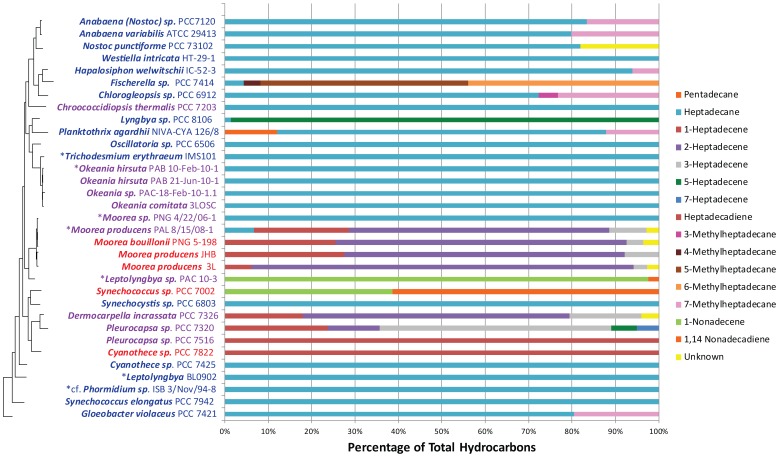
Hydrocarbon composition expressed as a percentage of total hydrocarbons for 32 strains of cyanobacteria displayed by phylogenetic relationship and pathway distribution. Percentages are displayed as mean percentage between three replicates except for those indicated with an asterisk for strains that were characterized using a single sample. Blue strain names indicate strains possessing the FAAR/ADO pathway and red indicates those with the OLS pathway. Purple strain names indicate a strain that does not have a genome sequence and therefore the pathway type is unknown. To the left of the figure, a 16S rRNA phylogenetic tree (Maximum Likelihood, see Figure S2 for complete tree) is presented for all 32 displayed strains. Branch tips are aligned to the corresponding strain names. Branches corresponding to *W. intricata* HT-29-1, *cf. Phormidium sp.* ISB 3/Nov/94-8 and *Pleurocapsa sp.* PCC 7320 are not shown because 16s rRNA sequences are not available for these strains. Vertical connection lengths were modified to accommodate the location of these three strains in the table.

Branched alkanes were observed in many of the strains investigated (7 of 32; Figure 4 and S2). Most commonly as 7-methylheptadecane, these branched alkanes were observed only in particular clades including heterocystous, ramified, and some filamentous cyanobacteria including *Planktothrix* (Figures 2–4). Additionally, 7-methylheptadecane was observed in this study from *Gloeobacter violaceus* PCC 7421, and constitutes a first report of branched hydrocarbons from this organism. *Fischerella sp.* PCC 7414 was found to produce several less common methylheptadecanes with the methyl groups assigned to positions C-4, C-5, and C-6 on the basis of fragmentation patterns (Figure 3 and S3). *Chlorogleopsis sp.* PCC 6912 also produces less commonly observed branched alkanes, namely 3- and 4-methylheptadecane (Figure 3). However, except for 3- and 7-methylheptadecanes for which authentic standards exist, definitive assignment of the position of methylation in the above alkanes remains tentative.

Through rigorous identification of double bond positions, this study was able to differentiate which strains produce terminal alkenes and which produce alkenes with double bonds at internal positions. Mass fragmentation of DMDS derivatives revealed several novel hydrocarbon structures such as 2- and 3-heptadecene (observed in all three *Moorea* strains as well as *Dermocarpella incrassata* PCC 7326) as well as the tentative identification of heptadecadiene in *Pleurocapsa sp.* PCC 7516.

**Figure 5:**
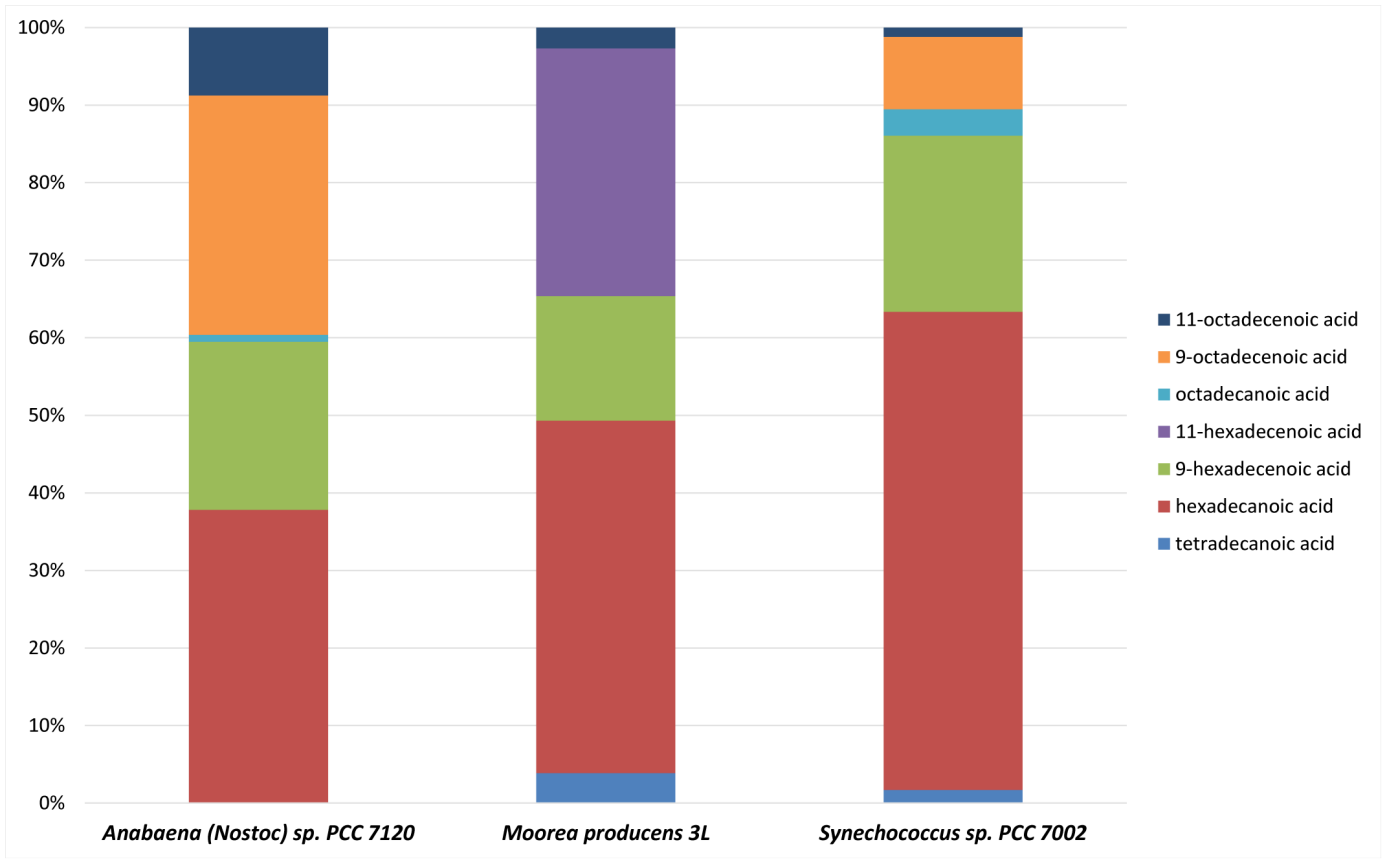
Fatty acid analysis of three cyanobacteria. Fatty acid analysis from single samples of A*nabaena (Nostoc) sp.* PCC 7120, *M. producens* 3L, and *Synechococcus sp.* PCC 7002. All three strains exhibit similar proportions of hexadecanoic acid and 9-hexadecenoic acid; however, A*nabaena (Nostoc) sp.* PCC 7120 exhibits a higher proportion of 9-octadecenoic acid and 11-octadecenoic acid and tetradecanoic acid is absent. *M. producens* 3L contains 11-hexadecenoic acid and no octadecanoic acid. *Synechococcus sp.* PCC 7002 exhibited a similar composition to A*nabaena (Nostoc) sp.* PCC 7120 yet has a higher proportion of hexadecanoic acid.

The fatty acids of *Anabaena (Nostoc) sp.* PCC 7120, *M. producens* 3L, and *Syenchococcus sp.* PCC 7002 were analyzed to evaluate the available substrates for alkane or alkene biosynthesis (Figure 5). Hexadecanoic acid was the most abundant fatty acid in all three strains followed by various unsaturated C16 and C18 fatty acids. *Anabaena (Nostoc) sp.* PCC 7120 possessed 9-hexadecenoic acid, 9- and 11-octadecenoic acid and octadecanoic acid in descending order of abundance. *M. producens* 3L also possessed 11-octadecenoic acid but in significantly lower abundance compared to 9- or 11-hexadecenoic acid, the two most abundant fatty acids after hexadecanoic acid. *M. producens* 3L also possessed tetradecanoic acid as well as 9-tetradecenoic acid. *Synechococcus sp.* PCC 7002 possessed 9-hexadecenoic acid in addition to 12-octadecenoic acid, 9, 12-octadecadienoic acid, octadecanoic acid and tetradecanoic acid in descending order of abundance. There was no evidence of odd chain length fatty acids in any of the strains investigated.

### Taxonomic Pathway Distribution

The recent addition of genome sequences from the CyanoGEBA project has more than doubled the amount of genomic information available for cyanobacteria and dramatically improved the distribution of genomic information across the phylogenetic diversity of cyanobacteria [43]. Prior to the availability of these sequences, only five cyanobacteria were known to possess the OLS pathway [*Synechococcus sp.* PCC 7002, *M. producens* 3L, *Cyanothece sp.* PCC 7822, *Cyanothece sp.* PCC 7424, and uncultured *Prochloron* symbionts [9], [21], [22]. Using the newly available CyanoGEBA data and newly sequenced genomes of *M. producens* JHB, *M. bouillonii* PNG5-198, *cf. Phormidium sp.* ISB 3/Nov/94-8, *P. agardii* NIVA-CYA 126/8, *H. welwitschii* IC-52-3, *W. intricata* HT-29-1 and *Leptolyngbya sp.* BL0902 (see experimental for sources), twelve additional strains were identified as possessing the OLS pathway, bringing the total to 17 strains (Figure 2). Nevertheless, the vast majority of genome-sequenced cyanobacteria possess the FAAR/ADO pathway (122 of 139). Interestingly, some of these latter cyanobacteria possess two homologs of the ADO gene (*Cyanothece sp.* PCC 7425, *Cyanobium gracile* PCC 6307, *Leptolyngbya sp.* PCC 7375, *Synechococcus sp.* CB0101, *Synechococcus sp.* RS9917). Strains that possess the OLS pathway are largely (14 of 17) within a single clade (Clade A), mostly comprised of strains from subdivisions II (formerly Pleurocapsales), subdivision III (formerly Oscillatoriales) and subdivision I (formerly Chroococcales). In addition, two strains in this study that fall into this clade (*D. incrassata* PCC 7326 and *Pleurocapsa sp.* PCC 7516) are not yet sequenced but produce terminal alkenes indicating that they likely also possess the OLS pathway. The two clades that are exceptions to this OLS pathway phylogenetic clustering are the clade containing *M. producens* 3L, *M. producens* JHB and *M. bouillonii* PNG5-198 as well as the more distant clade containing *Leptolyngbya sp.* PCC 6406 (Clade B &C, Figure 2). The clade containing the *Moorea* strains cannot be conclusively distinguished from the main OLS containing clade due to low bootstrap and posterior probability support, but *Leptolyngbya sp.* PCC 6406 is clearly in a distinct lineage. By 16S rRNA sequence, this freshwater filamentous strain clades with other *Leptolygnbya* strains as well as unicellular marine *Synechococcus* strains. Thus, *Leptolyngbya sp.* PCC 6406 is from a distinct evolutionary lineage from other OLS-containing cyanobacteria yet appears to possess this same metabolic pathway.

### Pathway Evolution

An alignment of DNA sequences for all known OLS pathways was constructed with annotations for open reading frames (Figure S4). This alignment also includes the CurM domain of the curacin A biosynthetic pathway, and as expected, shows that CurM does not contain the fatty acyl ACP ligase (FAAL) domain or the first acyl carrier protein (ACP) found in the OLS pathway (Figure S4). An alignment of the amino acid sequences of all 17 of the known OLS pathways and CurM shows that all of the OLS pathways contain the same domains and domain architecture (Figure S5). A phylogenetic tree of all OLS pathways was created to investigate their evolutionary relationships (Figure S6). This phylogenetic tree shows a similar topology with that produced from the corresponding 16S rRNA sequences (Figure 2) with the exception of *Leptolyngbya sp.* PCC 6406. In this latter case, the OLS sequence clades with those from the *Moorea* strains and CurM. A phylogenetic tree was generated for all available ADO genes as well; however, evolutionary relationships and topological comparisons to the 16S phylogenetic tree were prohibited due to poor bootstrap support (data not shown). Significantly, none of the genome sequenced cyanobacteria appear to contain both the FAAR/ADO pathway as well as the OLS pathway. Additionally, no pseudogenes with homology to either of the genes in the FAAR/ADO pathway were detected in OLS-containing cyanobacteria, and similarly, no pseudogenes were detected for the OLS pathway in strains containing the FAAR/ADO pathway.

## Discussion

A striking general conclusion that emerges from review of previous studies along with the results of this study is that hydrocarbon production is a universal phenomenon among cyanobacteria (Figure 2–4 and Table S1). However, hydrocarbon production in cyanobacteria appears to be derived from at least two very different pathways. Moreover, the specific structural features of the hydrocarbons reflect which pathway is present; saturated alkanes are found in strains with the FAAR/ADO pathway and terminal alkene containing-hydrocarbons are found only in OLS containing strains. As reported previously, heptadecane is the most commonly observed hydrocarbon in cyanobacteria followed by heptadecene, pentadecane and 7-methylheptadecane (Table S1) [2], [5], [44]. This observation is consistent with octadecanoic acid (FAAR/ADO) or hexadecanoic acid (OLS) precursor fatty acids, and these along with a variety of unsaturated derivatives are the most common fatty acids in cyanobacteria [45].

### Hydrocarbon Composition

Most of the strains investigated in this study had never been previously characterized for their hydrocarbon composition (22 of 32). This investigation found a wide variation in the content of hydrocarbons between these cyanobacterial strains with a range of only 0.024%±0.01% in *Cyanothece sp.* PCC 7425 to 0.262%±0.01% in *Pleurocapsa sp.* PCC 7516 (Figure 3). These results expand the previously reported range of natural cyanobacterial hydrocarbon yields that was between 0.025–0.12% dry weight [2], [4]. Strains possessing the OLS pathway appear to have significantly higher hydrocarbon yields (0.173%±0.032) than strains with the FAAR/ADO pathway (0.070%±0.008) (p-value = 0.0002).

Branched alkanes have been used as a biomarker for cyanobacteria, and consistent with this, they appear to be widely distributed across cyanobacterial phylogeny (Figure 2, Table S1) [2], [44]. Branched alkanes have been observed mostly in filamentous cyanobacteria but there are a few reports of branched hydrocarbons in unicellular strains (*Anacystis nidulans*, *Anacystis cyanea*, and *Chrocococcus turgidus*) [2], [30], [33]. Expanding this distribution, we show for the first time that the unicellular cyanobacterium *G. violaceus* PCC 7421 also produces 7-methylheptadecane. This study also expanded the known structural diversity of cyanobacterial hydrocarbons to include additional double bond positions (2- and 3-heptadecenes) and methyl group positions (3-, 4- and 5-methylheptadecanes). Overall, branched alkanes were observed in 7 of the 32 strains examined in this investigation. Branched alkane biosynthesis was previously investigated in cyanobacteria and the pendant methyl group found to be derived from S-adenosylmethionine (SAM) through a methyltransferase reaction [46], [47]. However, the methyltransferase involved in this pathway has yet to be identified and characterized. Han *et al.* [2] and Fehler & Light [46] used radiolabeled substrates to verify that vaccenic acid (11-octadecenoic acid) is the likely precursor to 7- or 8-methylheptadecane in *Nostoc muscorum*. We also observed 11-octadecenoic acid in our fatty acid analysis of *Anabaena (Nostoc) sp.* PCC 7120, thus confirming the possibility that this fatty acid is the precursor to these branched hydrocarbons in this strain (Figure 5).

All three *Moorea* strains as well as *D. incrassata* PCC 7326 produced the unique alkenes 2- and 3-heptadecene. Two possible pathways may be responsible for the production of these observed alkenes. First, a desaturase could act upon a fatty acid substrate to produce double bonds at the ω-2 or ω-3 position. The unsaturated fatty acid could then undergo elongation and decarboxylation via the OLS pathway followed by a single round of reduction to remove the newly introduced terminal double bond. Alternatively, an isomerase might act on 1-heptadecene to produce these 2- and 3-heptadecenes. Fatty acid analysis of *M. producens* 3L did not reveal any unsaturated fatty acids with double bonds at these ω-2 or ω-3 positions, but instead, hexadecanoic acid, 11-hexadecenoic acid, 9-hexadecenoic acid, and 11-octadecenoic were observed in order of decreasing abundance (Figure 5). The lack of ω-2 or ω-3 unsaturated fatty acids in this cyanobacterium suggests that an isomerase is likely involved in 2- and 3-heptadecene biosynthesis. Consistent with this hypothesis, the required 16∶0 fatty acid is the most abundant fatty acid in *M. producens* 3L.

### Phylogenetic Distribution

The phylogenetic distribution of the two known hydrocarbon biosynthetic pathways among cyanobacteria is revealing of their evolutionary history. The FAAR/ADO pathway is the most widely distributed pathway taxonomically (122 of 139 strains) and is therefore most likely to be the ancestral hydrocarbon pathway in cyanobacteria (Figure 2). Alternatively the OLS pathway is only found in a small number of cyanobacteria (17 of 139) and likely evolved later than the FAAR/ADO pathway (Figure 2). A striking finding revealed by this genomic investigation is the absence of the alternative hydrocarbon pathway in a strain when either the OLS or the FAAR/ADO pathway is present. More specifically, none of the cyanobacteria with the OLS pathway possess the FAAR/ADO pathway or any pseudogenes derived from it. In reciprocal fashion, none of the organisms that possess the FAAR/ADO pathway also possess the OLS pathway or any derived pseudogenes. Because the OLS pathway contains many of the same enzymatic domains found in PKS and fatty acid biosynthesis pathways (ACP, KS, AT, KR, TE), homologs for each domain or even groupings of domains can be found in all cyanobacteria. Thus, the distinctive domains of this pathway are the fatty acyl ACP ligase (FAAL), sulfotransferase (ST) and thioesterase (TE). These domains impart the specificity of the pathway to first utilize fatty acid substrates, activate the β-hydroxy fatty acid via sulfonation and then catalyze hydrolysis and decarboxylation to produce terminal alkenes. While homologous genes for each of these latter domains can be found throughout cyanobacterial phylogeny, the unique domain architecture that makes up the OLS pathway is found only in OLS containing cyanobacteria.

### Hydrocarbon Pathway Evolution

Most of the strains that possess the OLS pathway are found within a single clade on the 16S rRNA phylogentic tree (Clade A, Figure 2). Cyanobacterial phylogenies using multi-locus sequence analyses appear to exhibit the same placement of clade A within the tree topology [48]. However, this clade also includes many strains that possess the FAAR/ADO pathway, suggesting a unique evolutionary history that may have involved multiple horizontal gene transfer events. Thus, the evolutionary history of the OLS pathway in clade A cannot be definitively attributed to horizontal gene transfer without additional information including further genome sequences of OLS-containing cyanobacteria and phylogenomic comparisons.

The clade containing the *Moorea* strains (Clade B) as well as the separate clade containing *Leptolyngbya sp.* PCC 6406 (Clade C) are phylogenetically outside of this major clade of OLS containing cyanobacteria. The *Moorea* strains, however, cannot be definitively characterized as separate given the rather low bootstrap/posterior probability support values. Nevertheless, the topological placement of this group of filamentous tropical marine cyanobacteria in figure 2 is consistent with previous phylogenetic analyses [24]. Additionally, the OLS pathway found in the three genome sequenced strains of *Moorea* and in *Leptolyngbya sp.* PCC 6406 is consistently separated into two open reading frames with 9 to 17 bp intervening between the two ORFs (Figure S4). This distinctive pathway architecture is not found in any of the other 13 OLS-containing strains, and thus indicates a potentially distinct evolutionary history for these two groups of OLS pathways. Additionally, for all three *Moorea* strains and *Leptolyngbya sp.* PCC 6406, the full AA sequences from the OLS pathway, as well as the KS sequence considered separately, clade together and thus suggest a common evolutionary history (Figure S5 and S6). Despite the distinct pathway architectures and distinctive evolutionary history, the resulting hydrocarbon products do not appear to be fundamentally different as indicated by the production of 1-heptadecene in *Cyanothece sp.* PCC 7822 and the three *Moorea* strains.

*Leptolyngbya sp.* PCC 6406 contains the OLS pathway, but according to its 16S rRNA phylogeny, is very distantly related to the other strains containing the OLS pathway (Figure 2). This latter discrepancy between 16S rRNA and OLS gene tree topologies suggests that the OLS pathway in *Leptolyngbya sp.* PCC 6406 may have been obtained by horizontal gene transfer. Supporting this conclusion, the GC content of the *Leptolyngbya sp.* PCC 6406 OLS pathway (64%) is higher than that of the entire genome (55%); this contrasts with the OLS pathways in all other cyanobacteria which exhibit similar GC contents to their respective genomes (Table S3).

Thus, horizontal gene transfer appears to have played a role in the evolutionary history of the OLS pathway in cyanobacteria; however, the extent of this is unclear at this point. The competitive exclusion of the OLS and the FAAR/ADO pathway is intriguing and may suggest the presence of as yet unknown selective pressures to maintain one or the other of these hydrocarbon biosynthetic pathways, but not both. Further investigation of the physiological and ecological role of cyanobacterial hydrocarbons as well as further delineation of the phylogenetic distribution of these two pathways may reveal insights as to the nature of this selection and competitive exclusion pressure.

## Supporting Information

Figure S1 **Cyanobacterial 16S rRNA phylogeny and hydrocarbon pathway distribution for the compressed tree displayed in** **Figure 3** **.** The 16S rRNA phylogeny is displayed for the 20 cyanobacteria quantitatively characterized for their hydrocarbon composition. Blue strain names indicate strains possessing the FAAR/ADO pathway and red strain names indicate those with the OLS pathway. Purple strain names indicate a strain that does not have a genome sequence and therefore the pathway is unknown. Cyanobacterial subdivisions are labeled using colored branches following the key in the upper left: Subdivision I. Uniceullular (Formerly Chroococcales), Subdivision II. Baeocystous (Formerly Pleurocapsales), Subdivision III. Filamentous (Formerly Oscillatoriales), Subdivision IV. Heterocystous (Formerly Nostocales), Subdivision V. Ramified or True Branching (Formerly Stigonematales).
(TIF)

Figure S2 **Cyanobacterial 16S rRNA phylogeny and hydrocarbon pathway distribution for compressed tree displayed in** **Figure 4** **.** The 16S rRNA phylogeny is displayed for the 32 cyanobacteria characterized for their hydrocarbon composition as an overall percentage. Blue strain names indicate strains possessing the FAAR/ADO pathway and red strain names indicates those with the OLS pathway. Purple strain names indicate a strain that does not have a genome sequence and therefore the pathway is unknown. Cyanobacterial subdivisions are labeled using colored branches following the key in the upper left: Subdivision I. Uniceullular (Formerly Chroococcales), Subdivision II. Baeocystous (Formerly Pleurocapsales), Subdivision III. Filamentous (Formerly Oscillatoriales), Subdivision IV. Heterocystous (Formerly Nostocales), Subdivision V. Ramified or True Branching (Formerly Stigonematales).
(TIF)

Figure S3 **Mass spectra of branched hydrocarbons observed in** ***Fischerella sp.*** **PCC 7414.** The fragmentation patterns were used to propose the locations of methyl group substitutions on a heptadecane parent structure. In addition to the fragment losses depicted, neutral ion losses were also present.
(TIF)

Figure S4 **DNA alignment of all 17 known OLS pathways and CurM with annotated ORFs.** Four of the strains have the OLS pathway split into two ORFs. In these four strains the fatty acyl ACP ligase is a separate ORF from the PKS portion of the OLS pathway. Panel A shows all 17 aligned sequences with a red square highlighting the break between ORFs for the four pathways with two ORFs. Panel B shows the expanded red highlighted region from panel A. CurM is a part of the curacin A biosynthetic pathway which does not involve a FAAL [23].
(TIF)

Figure S5 **Amino acid alignment and phylogenetic tree of all 17 OLS pathways and the CurM domain.** All 17 of the OLS pathways contain the same domain architecture. CurM does not contain the FAAL and ACP1 domains. A maximum likelihood tree is displayed on the left of the alignment to depict the phylogenetic relationships between these pathways.
(TIF)

Figure S6 **Phylogeny of the KS domain for 17 cyanobacterial OLS pathways.** The KS domains from *Leptolyngbya sp.* PCC 6406 and the three *Moorea* strains clade together, suggesting a common evolutionary history.
(TIFF)

Table S1 **Summary of literature reports of cyanobacterial hydrocarbon composition.** Strains are organized by subdivision on the left-hand side, and a ‘+’ symbol indicates that a particular hydrocarbon was reported for this strain. The relevant reference is listed in the right-hand column with full references below. Findings that were validated using authentic standards or established using derivitization techniques to verify hydrocarbon structure are denoted with superscript 1.
(XLSX)

Table S2 **Sequence and Pathway Information.** Table of genes used in this investigation (16S, OLS, FAAR, ADO) with Genbank accession numbers and JGI IMG ID numbers. NA = Not Applicable, UA = Unavailable, UNK = Unknown.
(XLSX)

Table S3 **Genome and OLS gene percent GC for all 17 OLS pathway containing cyanobacteria.** Genome status is indicated (P-Permanent Draft, F-Finished, D-Draft). *Leptolyngbya sp.* PCC 6406 exhibits a higher GC content in its OLS pathway compared to its genome, as well as compared to other OLS pathways and their genomes.
(XLSX)
