# Supplementary figures and images for: Characterization of Cyanobacterial Hydrocarbon Composition and Distribution of Biosynthetic Pathways
Source: PLoS One. 2014 Jan 27;9(1):e85140. doi: 10.1371/journal.pone.0085140 (PMC3903477; doi:10.1371/journal.pone.0085140)

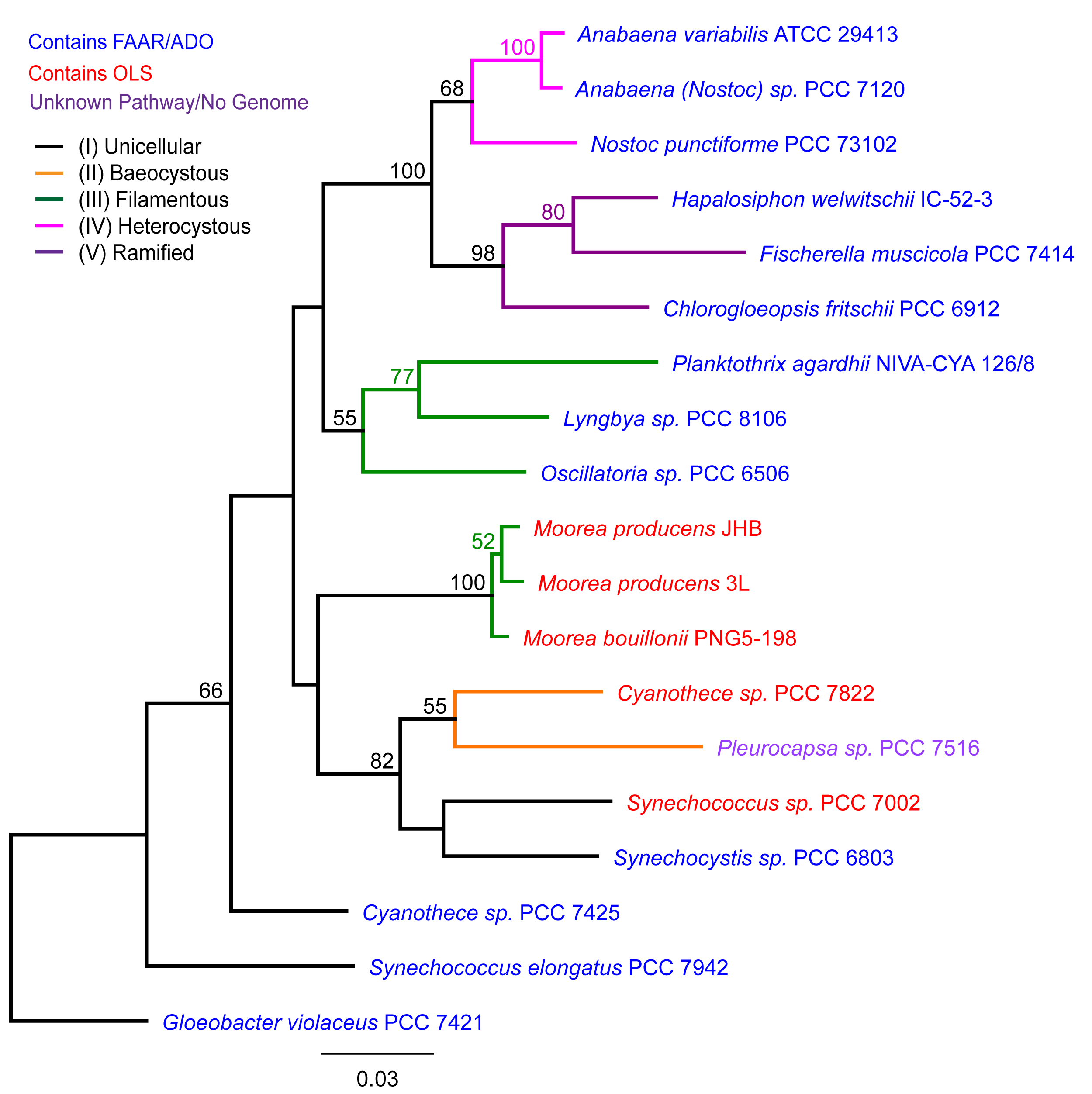

Supplement: Figure S1 — Cyanobacterial 16S rRNA phylogeny and hydrocarbon pathway distribution for the compressed tree displayed in Figure 3 . The 16S rRNA phylogeny is displayed for the 20 cyanobacteria quantitatively characterized for their hydrocarbon composition. Blue strain names indicate strains possessing the FAAR/ADO pathway and red strain names indicate those with the OLS pathway. Purple strain names indicate a strain that does not have a genome sequence and therefore the pathway is unknown. Cyanobacterial subdivisions are labeled using colored branches following the key in the upper left: Subdivision I. Uniceullular (Formerly Chroococcales), Subdivision II. Baeocystous (Formerly Pleurocapsales), Subdivision III. Filamentous (Formerly Oscillatoriales), Subdivision IV. Heterocystous (Formerly Nostocales), Subdivision V. Ramified or True Branching (Formerly Stigonematales). (TIF) [file pone.0085140.s001.tif]

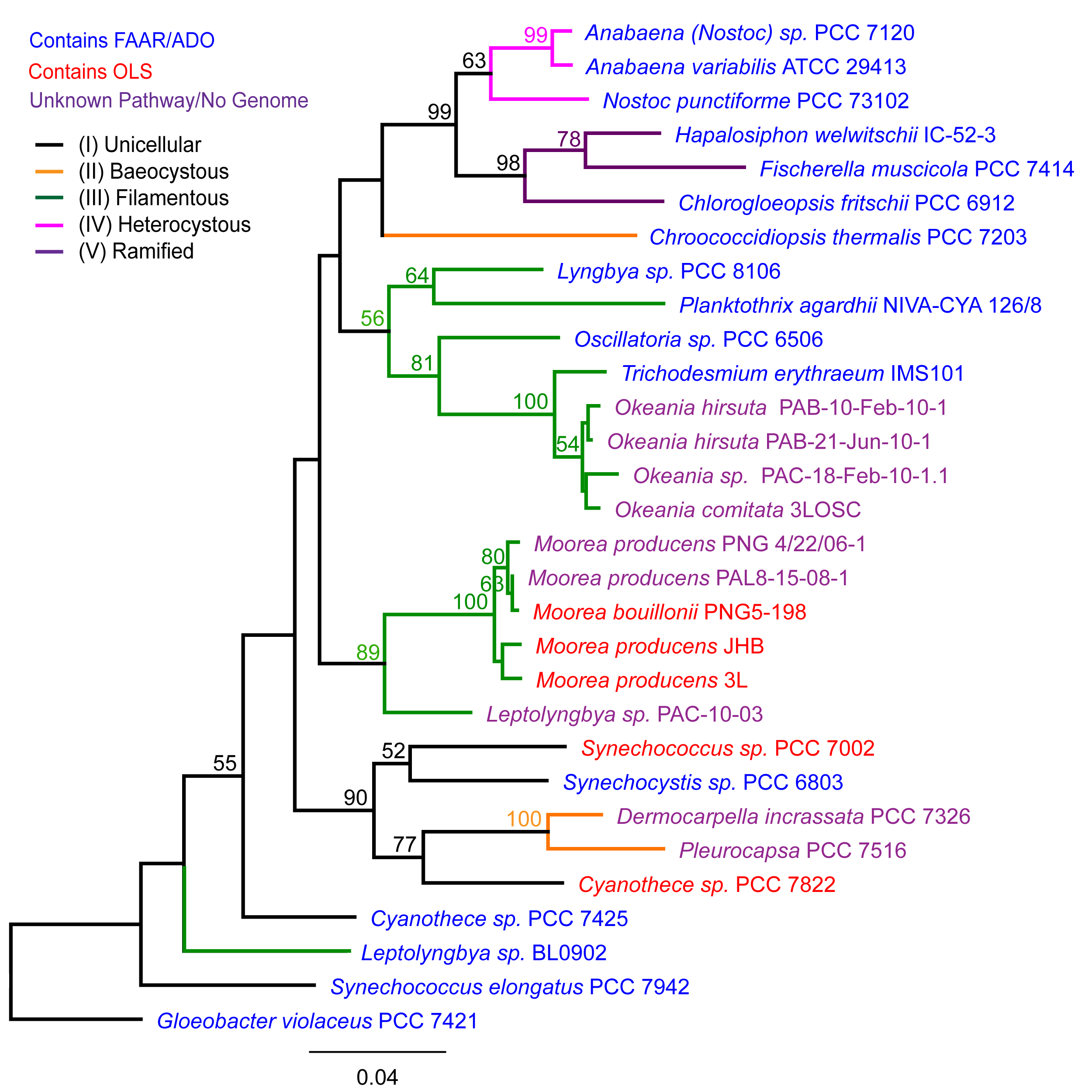

Supplement: Figure S2 — Cyanobacterial 16S rRNA phylogeny and hydrocarbon pathway distribution for compressed tree displayed in Figure 4 . The 16S rRNA phylogeny is displayed for the 32 cyanobacteria characterized for their hydrocarbon composition as an overall percentage. Blue strain names indicate strains possessing the FAAR/ADO pathway and red strain names indicates those with the OLS pathway. Purple strain names indicate a strain that does not have a genome sequence and therefore the pathway is unknown. Cyanobacterial subdivisions are labeled using colored branches following the key in the upper left: Subdivision I. Uniceullular (Formerly Chroococcales), Subdivision II. Baeocystous (Formerly Pleurocapsales), Subdivision III. Filamentous (Formerly Oscillatoriales), Subdivision IV. Heterocystous (Formerly Nostocales), Subdivision V. Ramified or True Branching (Formerly Stigonematales). (TIF) [file pone.0085140.s002.tif]

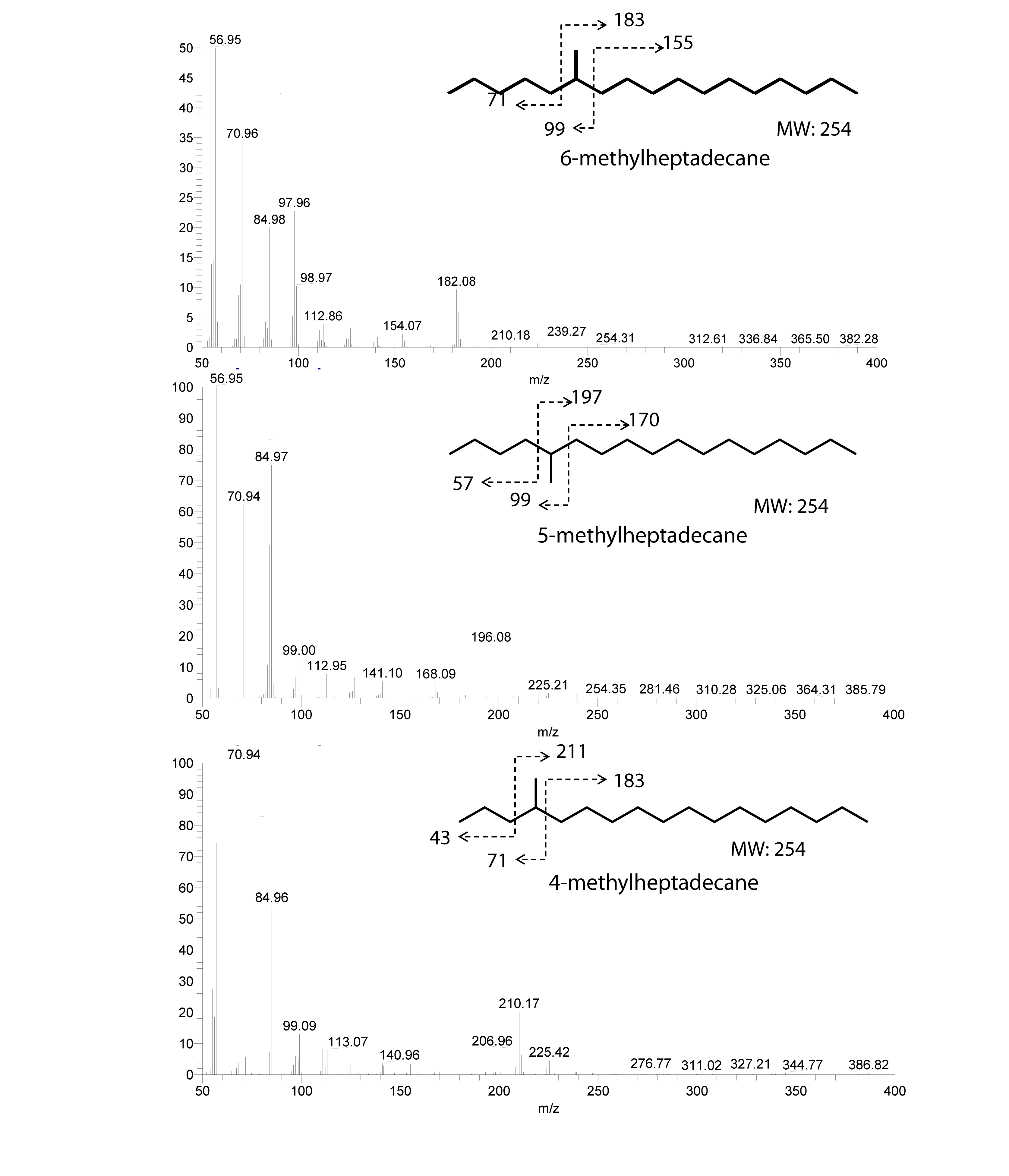

Supplement: Figure S3 — Mass spectra of branched hydrocarbons observed in Fischerella sp. PCC 7414. The fragmentation patterns were used to propose the locations of methyl group substitutions on a heptadecane parent structure. In addition to the fragment losses depicted, neutral ion losses were also present. (TIF) [file pone.0085140.s003.tif]

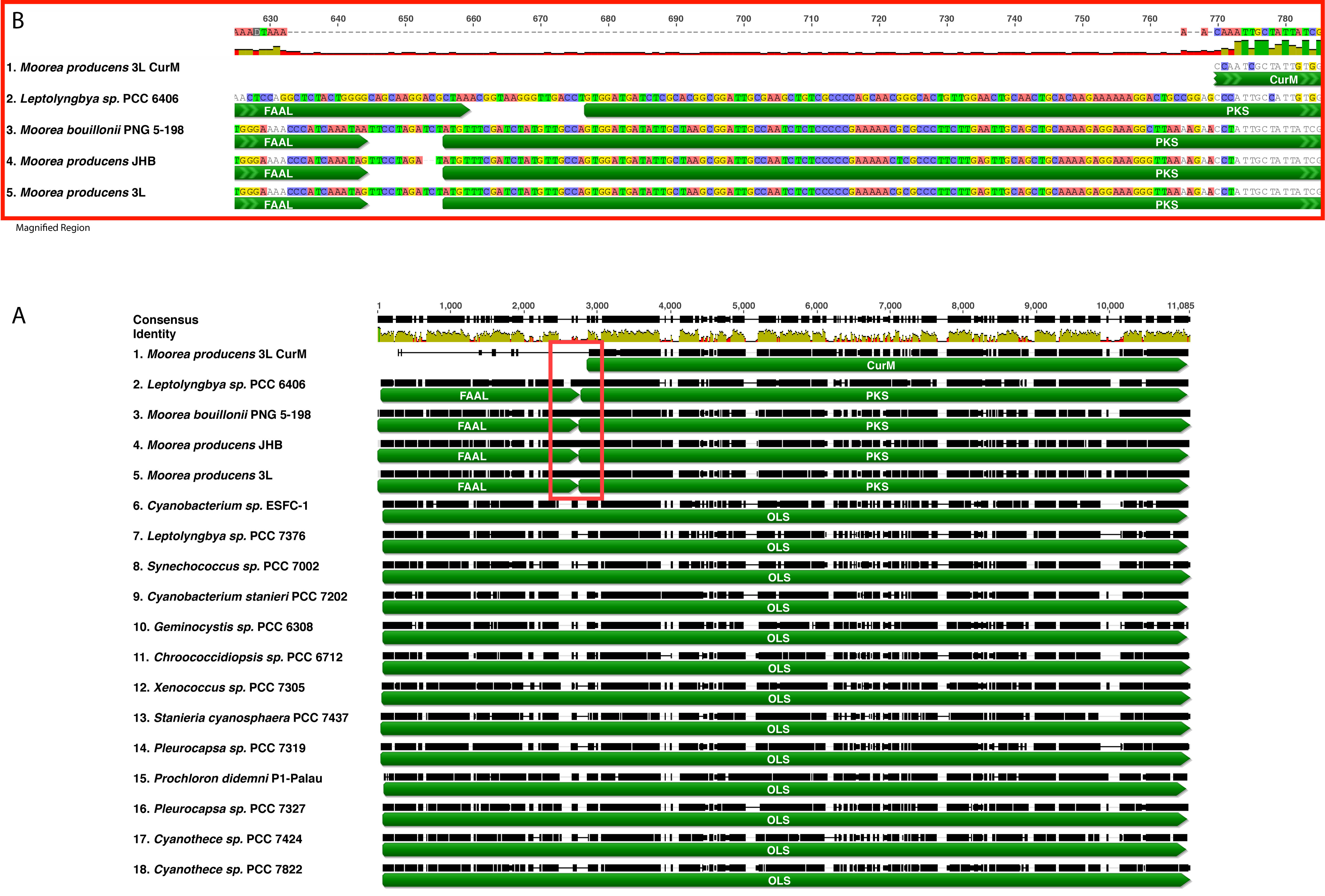

Supplement: Figure S4 — DNA alignment of all 17 known OLS pathways and CurM with annotated ORFs. Four of the strains have the OLS pathway split into two ORFs. In these four strains the fatty acyl ACP ligase is a separate ORF from the PKS portion of the OLS pathway. Panel A shows all 17 aligned sequences with a red square highlighting the break between ORFs for the four pathways with two ORFs. Panel B shows the expanded red highlighted region from panel A. CurM is a part of the curacin A biosynthetic pathway which does not involve a FAAL [23]. (TIF) [file pone.0085140.s004.tif]

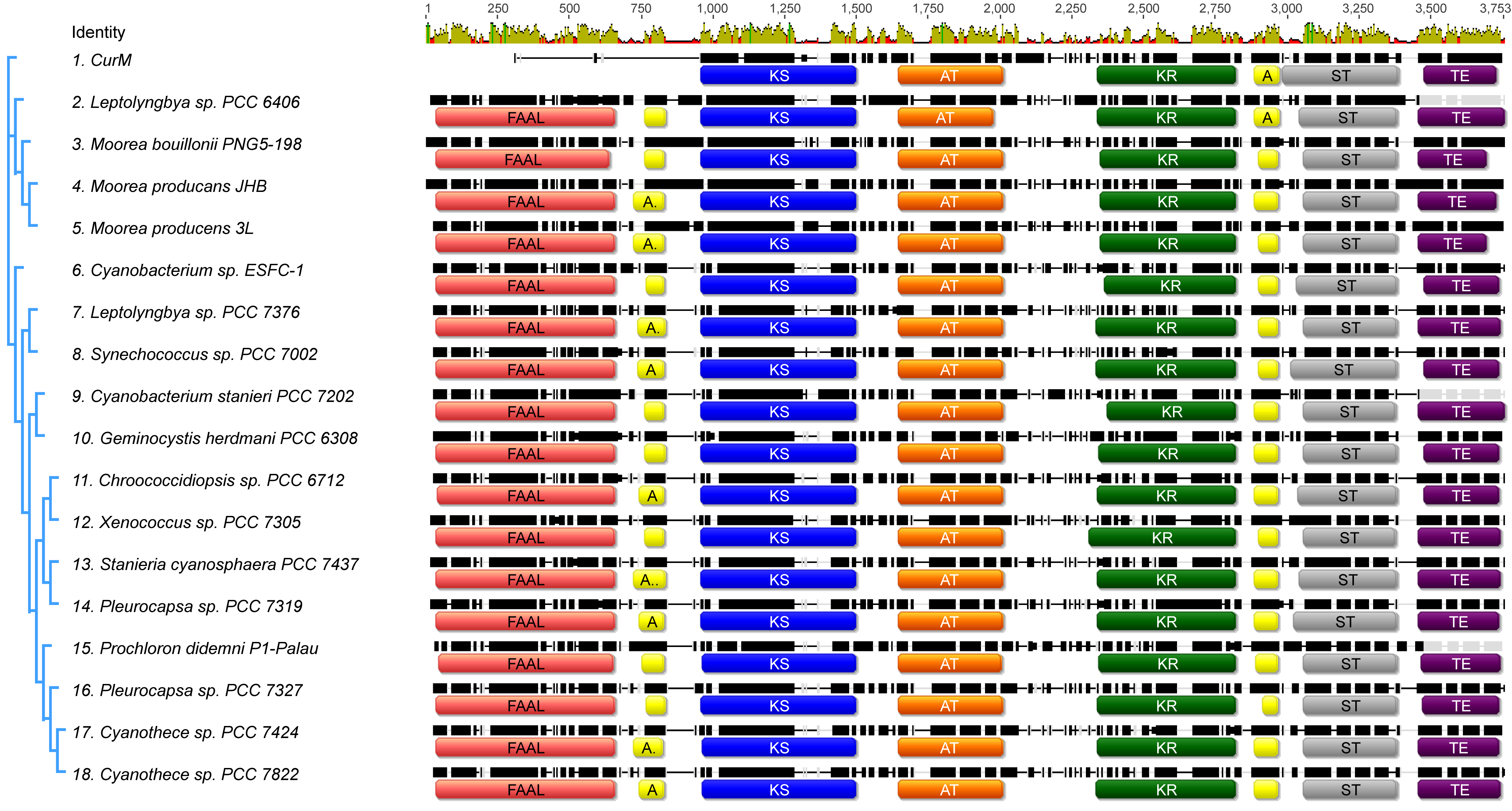

Supplement: Figure S5 — Amino acid alignment and phylogenetic tree of all 17 OLS pathways and the CurM domain. All 17 of the OLS pathways contain the same domain architecture. CurM does not contain the FAAL and ACP1 domains. A maximum likelihood tree is displayed on the left of the alignment to depict the phylogenetic relationships between these pathways. (TIF) [file pone.0085140.s005.tif]

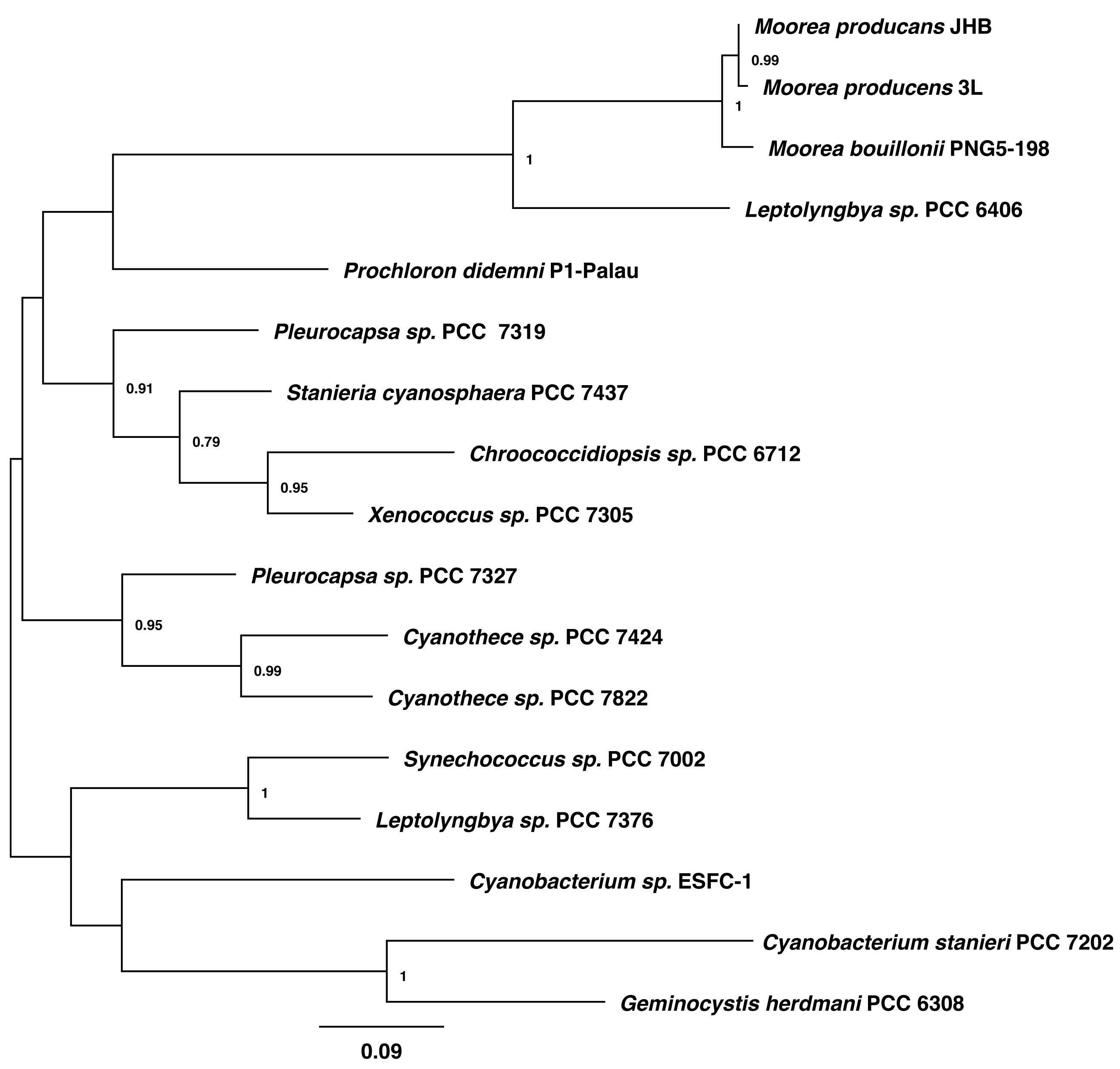

Supplement: Figure S6 — Phylogeny of the KS domain for 17 cyanobacterial OLS pathways. The KS domains from Leptolyngbya sp. PCC 6406 and the three Moorea strains clade together, suggesting a common evolutionary history. (TIFF) [file pone.0085140.s006.tiff]
